# Cognitive–behavioural versus cognitive–analytic guided self-help for mild-to-moderate anxiety: a pragmatic, randomised patient preference trial

**DOI:** 10.1192/bjp.2023.78

**Published:** 2023-09

**Authors:** Stephen Kellett, Charlotte Bee, Jess Smithies, Vikki Aadahl, Melanie Simmonds-Buckley, Niall Power, Caroline Dugen-Williams, Neil Fallon, Jaime Delgadillo

**Affiliations:** Rotherham Doncaster and South Humber NHS Foundation Trust, UK; and University of Sheffield, UK; Pennine Care NHS Foundation Trust, UK; Derbyshire Community Health Services NHS Foundation Trust, UK; Midlands Partnership NHS Foundation Trust, UK

**Keywords:** Anxiety disorders, randomised controlled trial, patient choice, cognitive–analytic therapy, improving access to psychological therapies

## Abstract

**Background:**

Guided self-help (GSH) for anxiety is widely implemented in primary care services because of service efficiency gains, but there is also evidence of poor acceptability, low effectiveness and relapse.

**Aims:**

The aim was to compare preferences for, acceptability and efficacy of cognitive–behavioural guided self-help (CBT-GSH) versus cognitive–analytic guided self-help (CAT-GSH).

**Method:**

This was a pragmatic, randomised, patient preference trial (Clinical trials identifier: NCT03730532). The Beck Anxiety Inventory (BAI) was the primary outcome at 8- and 24-week follow-up. Interventions were delivered competently on the telephone via structured workbooks over 6–8 (30–35 min) sessions by trained practitioners.

**Results:**

A total of 271 eligible participants were included, of whom 19 (7%) accepted being randomised and 252 (93%) chose their treatment. In the preference cohort, 181 (72%) chose CAT-GSH and 71 (28%) preferred CBT-GSH. BAI outcomes in the preference and randomised cohorts did not differ at 8 weeks (−0.80, 95% confidence interval (CI) −4.52 to 2.92) or 24 weeks (0.85, 95% CI −2.87 to 4.57). After controlling for allocation method and baseline covariates, there were no differences between CAT-GSH and CBT-GSH at 8 weeks (F(1, 263) = 0.22, *P* = 0.639) or at 24 weeks (*F*(1, 263) = 0.22, *P* = 0.639). Mean BAI change from baseline was a reduction of 9.28 for CAT-GSH and 9.78 for CBT-GSH at 8 weeks and 12.90 for CAT-GSH and 12.43 for CBT-GSH at 24 weeks.

**Conclusions:**

Patients accessing routine primary care talking treatments prefer to choose the intervention they receive. CAT-GSH expands the treatment offer in primary care for patients with anxiety seeking a brief but analytically informed GSH solution.

## Guided self-help

Guided self-help (GSH) is an empirically supported intervention for mild-to-moderate anxiety and depression.^1^ GSH is a brief and low-intensity psychoeducational intervention of 6–8, 30–35 min sessions.^2^ GSH is facilitated by trained and well supervised practitioners and has been shown to consistently outperform unguided self-help.^3^ GSH is routinely delivered in primary mental healthcare systems globally^4^ and is one of the defining features of the Improving Access to Psychological Therapies (IAPT) programme in England (now called NHS Talking Therapies for Anxiety and Depression), where many patients are treated with this approach annually.^5^ However, GSH based on cognitive–behavioural therapy (CBT-GSH) is unacceptable to many patients, who drop-out early and attain poorer treatment outcomes.^6^

## Patient choice

Supporting personal preferences regarding what type and how treatment is delivered is enshrined in health policy.^7^ However, patients complain that choice is often either not initially elicited or is subsequently ignored in routine services.^8^ Additionally, when preferences are not taken into consideration during clinical trials, low participation rates can occur which then limits generalisability.^9^ Meta analyses show that preference accommodation can reduce treatment refusal rates, drop-out and loss to follow-up,^10^ but the relationship with treatment outcome is less clear.^11^ Partially randomised patient preference trials (PRPPT) offer a methodological solution, as participants with strong treatment preferences receive their treatment of choice and those without strong preferences are randomised.^9^

## Current study

To fill the gap in the evidence base concerning the acceptability and efficacy of GSH, we conducted a PRPPT of two versions of GSH (CBT-GSH versus cognitive–analytic therapy GSH (CAT-GSH)) for patients meeting diagnostic threshold for an anxiety disorder. This study tested four hypotheses:

- similar proportions of patients would express a treatment choice for each intervention;
- participants choosing their treatments would have significantly better outcomes;
- there would not be statistically significant between-group differences at 8- and 24-week follow-up on the primary outcome measure for anxiety;
- there would not be statistically significant differences between interventions on secondary outcomes.

## Method

### Study design

This study was conducted in an NHS Talking Therapies for Anxiety and Depression service in northern England and patients were first recruited on 29 January 2019. The service is part of the national IAPT programme delivering evidenced-based psychological interventions for anxiety and depression in a stepped-care model.^5^ This study followed a PRPPT design and had four arms:

- preference allocation to CAT-GSH (arm 1) or
- preference allocation to CBT-GSH (arm 2) or
- random allocation to CAT-GSH (arm 3) or
- random allocation to CBT-GSH (arm 4).

All procedures contributing complied with the ethical standards of the relevant national and institutional committees on human experimentation and with the Helsinki Declaration of 1975, as revised in 2008.^12^ All procedures involving human patients were approved by the Health Research Authority (240751) and a full protocol was published and followed.^13^ All protocol amendments were approved by the NHS Research Ethics Committee and were typically related to the COVID-19 pandemic (see Supplementary Appendix 1 available at https://doi.org/10.1192/bjp.2023.78). This study followed CONSORT reporting guidelines. Clinical trials identifier: NCT03730532.

### Participants

Patients were included in the study when they:

- were referred by a general practitioner;
- met criteria for an anxiety disorder based on the Mini International Neuropsychiatric Interview (MINI);^14^
- met ‘caseness’ on the Beck Anxiety Inventory (i.e. BAI ≥10);^15^
- were willing to engage in GSH;
- could attend 6–8 sessions; and
- were ≥18 years old.

Patients were excluded when they:

- were already accessing therapy;
- did not meet MINI criteria for an anxiety disorder;
- did not screen positive on the BAI;^15^
- met criteria for depression and a comorbid anxiety disorder, where depression was more severe;
- had a severe/chronic mental health problem and were involved in secondary care mental health services;
- had a diagnosis of social phobia or post-traumatic stress disorder, as IAPT guidelines^16^ recommend traditional psychotherapies for these conditions;
- GSH sessions would require an interpreter; and
- were unable to read and write.

Written informed consent was obtained from all participants. Participants involved in secondary care because of a severe/chronic mental health problem were excluded as the study was set in an NHS Talking Therapies for Anxiety and Depression service. The nature of the interventions being tested in this study were designed for delivery with mild-to-moderate anxiety. Participants were excluded whose GSH sessions would require an interpreter because the workbooks were only available in English.

### Preference, randomisation and masking

At the research screening interview, patient preferences were elicited using a detailed treatment information sheet. CBT-GSH was described as working purely in the here-and-now, having a focus on thought–feeling–behaviour linkages, using homework exercises and making less use of the therapeutic relationship. Although CBT-GSH relies on good engagement skills and the ability to build therapeutic alliances, it does not analyse the therapeutic relationship as a change method. CAT-GSH was described as working with the past and present, working with the therapeutic relationship and those dynamics, making use of homework exercises, and taking an explicitly relational approach.

Participants that did not state a treatment preference were computer allocated to either CAT-GSH or CBT-GSH using parallel group 1:1 randomisation by an independent researcher not directly involved in the treatment of study participants. Participants and practitioners were not masked to treatment allocation, but researchers collecting outcomes at follow-up were masked to treatment allocation.

### Procedures

#### Assessment interviews

Participants were recruited in two stages. An initial assessment was conducted on the telephone for 40 min by qualified psychological well-being practitioners (PWPs), following IAPT practice guidelines,^16^ including psychometric assessment. When anxiety scores measured by the Generalised Anxiety Disorder-7 Questionnaire (GAD-7)^17^ met ‘caseness’ (i.e., were ≥8), a research eligibility screening interview was then offered. A script was used to ensure the study was introduced without bias for either intervention. Trained research staff conducted this second screening using the MINI^14^ and the BAI.^15^ At these interviews, treatment or randomisation preferences were elicited.

#### Psychological interventions

Trial interventions were delivered by *n* = 16 qualified PWPs and involved 6–8 (30–35 min) weekly telephone sessions of one-to-one GSH. All PWPs had passed an IAPT 1-year post-graduate certificate in CBT-GSH following a national curriculum. All PWPs attended 2-day CAT-GSH training. Interventions were guided by structured workbooks, containing psychoeducation, in-session exercises and homework exercises for both CBT-GSH and CAT-GSH. The psychoeducation, in-session exercises and homework exercises differed between CAT-GSH and CBT-GSH according to the underlying theory and approach. CAT-GSH also differed from CBT-GSH in that exercises contained in the workbook allowed the dyad to analyse the therapeutic relationship for evidence of when the roles and patterns described in the workbook were also being enacted in the therapeutic relationship. CAT-GSH has been previously found to produce effective and durable anxiety outcomes,^18^ with PWPs finding it a highly acceptable form of GSH that markedly differs in approach to CBT-GSH.^19^ PWPs had 1 h per week of individual case management supervision and were enrolled in group supervision once a month for 2 h for each version of GSH.

The validated six-item low-intensity treatment competency scale (LITC)^20^ was used to assess GSH treatment competency, where a score of ≥18 defines competent GSH. One randomly selected session was audio-recorded per participant. Some participants dropped out prior to the selected session, so *n* = 94 (*n* = 70 CAT-GSH; *n* = 24 CBT-GSH) recordings were available. All these sessions were first rated by a trained independent rater using the LITC. Two teams of expert PWP raters, three in each team, in a fully crossed design, then rated six sessions drawn equally from CAT-GSH and CBT-GSH and so rated 12 sessions overall. Sessions were selected to have a range of competency according to the first independent rater's LITC score, but were blind rated by the expert PWPs. Indices of interrater reliability ranged between 0.85 and 0.99. The mean LITC score for all the recorded sessions was 19.34 (s.d. 2.85) for CAT-GSH and 19.94 (s.d. 2.92) for CBT-GSH; competency scores did not differ (*t*(92) = 1.20, *P* = 0.584).

### Measures

The primary outcome was anxiety severity measured by the BAI at 8- and 24-weeks follow-up, adjusted for baseline severity. The BAI has been extensively used and tested and found to be a valid and reliable index of anxiety severity.^15^ A reliable change on the BAI is a change score of ≥10 points and the caseness cut-off score is a score of 10. Rates of reliable change and reliable and clinically significant change (RCSC) on the BAI were calculated at each follow-up point.

Secondary measures included the Patient Health Questionnaire-9,^21^ GAD-7^17^ and Work and Social Adjustment Scale (WSAS).^22^ These were completed at initial assessments, at each treatment session, and at 8 and 24-week follow-up. Outcome definitions for these measures (i.e. reliable recovery, recovery, reliable improvement and reliable deterioration) followed IAPT criteria.^16^ The most stringent outcome definition of ‘reliable recovery’ requires RCSC on both the PHQ-9 and GAD-7. Outcomes were benchmarked against the routine outcomes achieved in the service (i.e. drawn from non-trial patients) for the duration of the PRPPT. As a result of the pragmatic nature of this PRPPT, the number of participants that had accessed the IAPT service prior to and following the trial was also assessed. Other secondary outcomes included the adverse incident rate, the number of sessions attended, drop-out and stepping-up rates (i.e. percentages of participants stepped up to traditional psychotherapies because of lack of response to GSH).

### Statistical analysis

The analysis followed an established PRPPT analytic approach set out in the study protocol.^13^ First, any differences between randomised and preference groups within each of the two GSHs were assessed. If no systematic differences were observed then these were collapsed to form a two-arm trial (i.e., CBT-GSH versus CAT-GSH). The *a priori* sample size calculation required a minimum of *n* = 134 participants for the primary analysis.^13^ Primary and secondary outcomes were analysed following intention-to-treat (ITT) principles, including all allocated participants. The two final ITT samples comprised: (a) a randomised cohort versus a preference cohort and (b) a CBT-GSH cohort versus a CAT-GSH cohort. Missing data were imputed using missForest for each treatment group separately based on all available demographic and clinical measures. Sensitivity analyses were conducted repeating the statistical analyses in the complete case sample (participants with complete data at all three time points).

The statistical analysis plan included: (a) producing a CONSORT summary; (b) calculating between-group Cohen's *d* effect sizes on the BAI at 8- and 24-weeks follow-up; (c) comparing RCSC rates on the BAI and secondary measures in each arm at 8- and 24-week follow-up; (d) examining between-arm differences on primary and secondary outcomes using analysis of covariance (ANCOVA). To account for potential confounding variables in the combined two-arm treatment comparison, treatment allocation and preference variables were included as covariates in the ANCOVA analyses. To test the robustness of the results, the analysis was repeated using longitudinal multilevel modelling, where repeated outcome measures (level 1) were nested within case participants (level 2), with mean-centred continuous variables, controlling for baseline severity and introducing a group variable along with a group × time interaction term (the latter being the primary hypothesis test). All analyses were carried out by a researcher masked to group allocation, and were conducted in R using *missforest*, *car*, *emmeans* and *compute.es*.

## Results

**Fig. 1:**
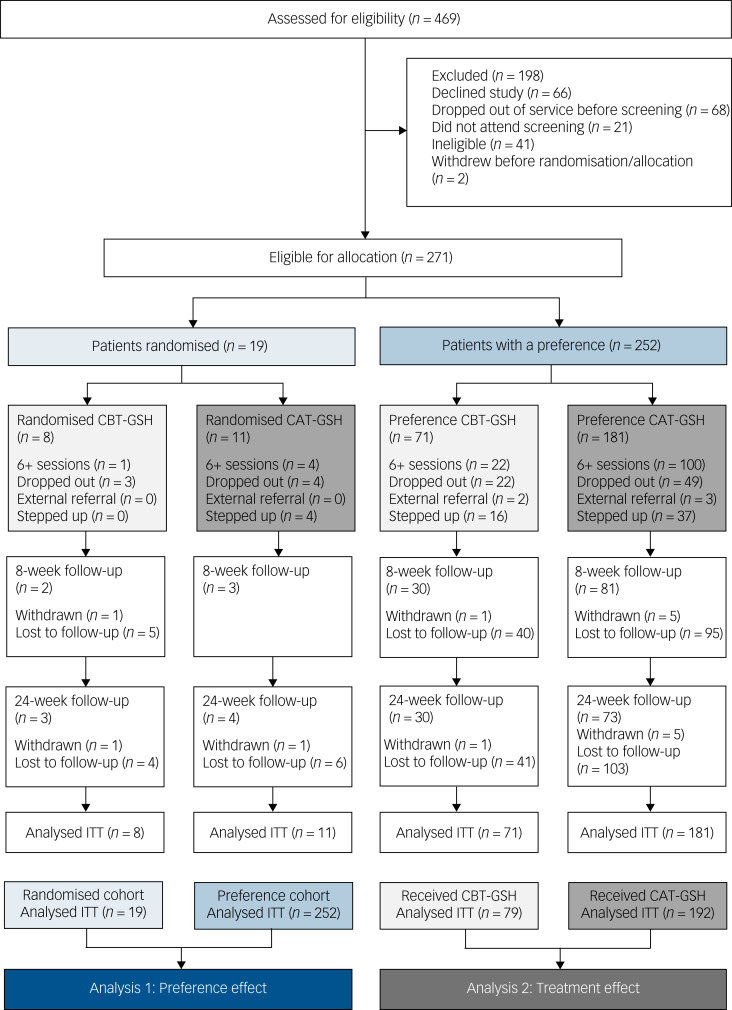
CONSORT flowchart of trial sample (CBT-GSH, cognitive–behavioural therapy guided self-help; CAT-GSH, cognitive–analytic therapy guided self-help).

The CONSORT diagram is presented in Fig. 1. In total, 469 patients were assessed for eligibility. Of the 271 eligible participants, 19 (7%) were randomised and 252 (93%) received their treatment of choice. In the preference allocation cohort (i.e. *n* = 252), then 71 (28%) chose CBT-GSH and 181 (72%) chose CAT-GSH.

### Participant description

Baseline comparisons were examined in relation to (a) randomised versus preference cohorts, (b) randomised versus preference cohorts nested within each treatment arm, and (c) the overall CBT-GSH versus CAT-GSH interventions. All comparisons indicated similarity across groups (see Table 1 and Supplementary Appendix 2). Therefore, a two-arm comparison of CBT-GSH versus CAT-GSH was conducted.^19^

**Table 1:** Baseline characteristics for the randomised versus preference cohorts; the CBT-GSH versus CAT-GSH conditions; and the total cohort^a^

	Randomised (*n* = 19)	Preference (*n* = 252)	Between-group difference, *P*	CBT-GSH (*n* = 79)	CAT-GSH (*n* = 192)	Between-group difference, *P*	Total (*n* = 271)
Demographic characteristics							
Age, years, mean (s.d.)	38.47 (16.22)	36.62 (13.74)	0.575	36.01 (13.43)	37.05 (14.11)	0.579	36.75 (13.90)
Sex, *n* (%)			0.790			**0.013**	
Male	5 (26)	62 (25)		28 (35)	39 (20)		67 (25)
Female	14 (74)	190 (75)		51 (65)	153 (80)		204 (75)
Ethnicity, *n* (%)			1.00			0.481	
White	18 (95)	228 (90)		73 (92)	173 (90)		246 (91)
Black and minority ethnic	1 (5)	23 (9)		5 (6)	19 (10)		24 (9)
Missing	0 (0)	1 (0.4)		1 (1)	0 (0)		1 (0.4)
Employment status, *n* (%)			1.00			0.701	
Unemployed	2 (11)	35 (14)		12 (15)	24 (13)		37 (14)
Employed/other	15 (79)	214 (85)		67 (85)	162 (84)		229 (85)
Missing	2 (11)	3 (1)		0 (0)	5 (3)		5 (2)
LTC, *n* (%)			0.619			1.00	
Self-report LTC	8 (42)	87 (35)		28 (35)	67 (35)		95 (35)
No LTC	11 (58)	165 (65)		51 (65)	125 (65)		176 (65)
Taking medication			0.146			0.411	
Taking	13 (68)	129 (51)		45 (57)	97 (51)		142 (52)
Not taking	5 (26)	112 (44)		31 (39)	86 (45)		117 (43)
Missing	1 (5)	11 (4)		3 (4)	9 (5)		12 (4)
Previous treatment history, *n* (%)							
Previous treatment disclosed	7 (37)	108 (43)	0.641	20 (25)	95 (50)	**<0.001**	115 (42)
Had CBT	3 (16)	73 (29)	0.293	12 (15)	64 (33)	**0.003**	76 (28)
Had CAT	0 (0)	3 (1)	1.00	0 (0)	3 (2)	0.558	3 (1)
Outcome measures							
BAI score, mean (s.d.)	31.63 (11.80)	26.10 (10.22)	**0.025**	28.10 (10.30)	25.81 (10.41)	0.101	26.49 (10.41)
Mild, *n* (%)	1 (5)	44 (18)	0.386	10 (13)	35 (18)	0.424	45 (17)
Moderate, *n* (%)	7 (37)	79 (31)		24 (30)	62 (32)		86 (32)
Severe, *n* (%)	11 (58)	129 (51)		45 (57)	95 (50)		140 (52)
GAD-7 score, mean (s.d.)	16.95 (3.75)	16.00 (3.89)	0.307	16.86 (3.44)	15.74 (4.00)	**0.031**	16.07 (3.88)
PHQ-9 score, mean (s.d.)	16.53 (3.91)	15.42 (5.54)	0.396	16.87 (5.01)	14.94 (5.52)	**0.008**	15.50 (5.44)
WSAS score, mean (s.d.)	19.78 (8.65)	20.15 (8.19)	0.854	21.35 (7.41)	19.61 (8.47)	0.112	20.12 (8.20)

*P-*values significant at <0.05 are highlighted in bold. BAI, Beck Anxiety Inventory; CAT-GSH, cognitive–analytic therapy-guided self-help; CBT-GSH, cognitive–behavioural therapy guided self-help; GAD-7, Generalised Anxiety Disorder-7; LTC, long-term condition (defined as a comorbid long-term physical health condition); PHQ-9, Patient Health Questionnaire-9; WSAS, Work and Social Adjustment Scale.

a. Between-group differences are based on independent *t*-tests for continuous variables and chi-squared tests for categorical variables.

In these combined cohorts, CBT-GSH participants were significantly more likely to be male, had not received any previous psychological intervention and had more severe anxiety and depression symptoms at baseline (see Table 1). Overall, twice as many patients who received CAT-GSH had received a previous psychological intervention, with 33% having previously received a CBT-based intervention in the service. These observed differences between the GSH treatments, combined with the PRPPT design (i.e. the high proportion of patients stating a treatment preference) indicated the need to adjust for baseline variables significantly associated with allocation (i.e. preference versus randomised) and intervention (i.e. CBT-GSH versus CAT-GSH).

The adjusted ANCOVA models of the primary and secondary treatment effects controlled for baseline differences in BAI, PHQ-9 and GAD-7 severity, gender, previous treatment history and allocation choice. The baseline-severity-only-adjusted analyses without the additional covariates are reported in the Supplementary Appendix 3 for comparison.

### Preference effect

The high treatment preference rate (93%) compared with randomisation rate (7%) meant that tests of the preference-accommodation effect were exploratory (for full analyses, see Supplementary Appendix 3). There were no significant differences in BAI scores between the preference and randomised cohorts at 8-week (−0.80; 95% CI −4.52 to 2.92; *P* = 0.672; *d* = 0.20) or 24-week follow-up (0.85; 95% CI −2.87 to 4.57; *P* = 0.626; *d* = 0.21), controlling for baseline severity. Comparisons of attendance, drop-out and lost-to-follow-up rates between the randomised and preference cohorts suggested an overall pattern of better attendance and engagement in the preference cohort, although most differences were not statistically significant, except for significantly greater rates of attendance for the preference cohort in the total and CAT-GSH samples (see Supplementary Tables 2 and 3 in Appendix 4).

### Treatment effect

#### Primary outcome

Table 2 reports the primary outcomes. At baseline, the BAI scores for the *n* = 271 participants included in the ITT analysis were 28.10 (s.d. = 10.30) for the CBT-GSH and 25.81 (s.d. = 10.41) for the CAT-GSH. Between-group differences in adjusted mean BAI scores at 8-week follow-up were not statistically significant (*F*(1, 263) = 0.22, *P* = 0.639). The mean difference slightly favoured CBT-GSH (Table 2; 0.50, 95% CI −1.61 to 2.61), representing a minimal Cohen's *d* effect size (*d* = 0.06, 95% CI −0.19 to 0.30). These small and non-significant differences between the GSH interventions were supported by the complete case analysis (*F*(1, 106) = 0.07, *P* = 0.797), where the adjusted between-group difference on the BAI of 0.57 (−3.81 to 4.94) also favoured CBT-GSH (Supplementary Appendix 5; *d* = 0.05, 95% CI −0.34 to 0.45).

**Table 2:** Primary and secondary outcome measures at post-treatment (8 weeks) and follow-up (24 weeks) in the ITT and complete case samples

	CAT-GSH *N* = 192	CBT-GSH *N* = 79	ITT analysis (observed and imputed data)^a^	Complete case analysis (observed data only)^b^
	Mean change from baseline^c^ (s.e)	Mean change from baseline^c^ (s.e)	Adjusted between-group difference^d^ (95% CI)	Adjusted between-group difference^d^ (95% CI)
Primary outcome				
BAI				
8-week post-treatment	9.28 (1.03)	9.78 (1.18)	0.50 (−1.61 to 2.61)	0.57 (−3.81 to 4.94)
24-week follow-up	12.90 (0.96)	12.43 (1.10)	−0.47 (−2.43 to 1.50)	−1.28 (−5.41 to 2.85)
Secondary outcomes				
GAD-7				
8-week post-treatment	6.22 (0.55)	5.84 (0.62)	−0.39 (−1.51 to 0.73)	0.49 (−1.80 to 2.78)
24-week follow-up	6.98 (0.65)	6.41 (0.73)	−0.57 (−1.67 to 0.53)	−0.38 (−2.67 to 1.91)
PHQ-9				
8-week post-treatment	4.96 (0.59)	4.81 (0.68)	−0.15 (−1.36 to 1.06)	0.87 (−1.70 to 3.44)
24-week follow-up	5.69 (0.56)	5.35 (0.64)	−0.34 (−1.48 to 0.81)	−0.25 (−2.62 to 2.13)
WSAS				
8-week post-treatment	4.26 (0.92)	4.67 (1.06)	0.41 (−1.49 to 2.30)	2.25 (−1.83 to 6.32)
24-week follow-up	5.53 (0.89)	5.90 (1.01)	0.37 (−1.46 to 2.20)	0.21 (−3.63 to 4.05)

BAI, Beck Anxiety Inventory; CAT-GSH, cognitive–analytic therapy-guided self-help; CBT-GSH: cognitive–behavioural therapy-guided self-help; GAD-7, Generalised Anxiety Disorder-7; ITT, intention-to-treat; PHQ-9, Patient Health Questionnaire-9; WSAS, Work and Social Adjustment Scale; s.e., standard error.

a. ITT sample based on missing outcome data imputed using missForest.

b. Observed data based on *n* = 84 in CAT-GSH and *n* = 32 in CBT-GSH at 8 weeks; *n* = 77 in CAT-GSH and *n* = 33 in CBT-GSH at 24 weeks.

c. Estimated marginal mean change scores from baseline are reported for the ITT sample.

d. Scores are adjusted for BAI baseline severity, allocation choice (preference vs. randomised), gender, previous treatment, GAD-7 baseline severity and PHQ-9 baseline severity. Analysis of WSAS outcomes are also adjusted for WSAS baseline severity. Continuous covariates are mean centred.

There were no statistically significant differences at 24-weeks follow-up (*F*(1, 263) = 0.22, *P* = 0.639), with a mean BAI difference of −0.47 (95% CI −2.43 to 1.50) favouring CAT-GSH (*d* = 0.05, 95% CI −0.20 to 0.29). This was replicated in the complete-cases analysis (*F*(1, 98) = 0.38, *P* = 0.541), but with a mean difference of 1.28 BAI points favouring CAT-GSH (*d* = 0.12, 95% CI −0.27 to 0.51).

#### Secondary outcomes

Secondary clinical outcomes are reported in Tables 2 and 3. Sensitivity analyses using longitudinal multilevel modelling found no significant effect of the time × treatment group interaction (full results are reported in Supplementary Appendix 6). This suggests no significant differences in the longitudinal symptom trajectories observed during both GSH interventions. BAI reliable change rates were 50.63% for CBT-GSH and 38.54% of CAT-GSH at 8-weeks follow-up, which were not significantly different (*x*^2^ = 2.90, *P* = 0.090). Rates increased to 65.82% (CBT-GSH) and 56.77% (CAT-GSH) at 24-week follow-up, with differences remaining non-significant (*x^2^* = 1.50, *P* = 0.214). Using the more stringent RCSC criteria on the BAI at 8 weeks, there was no difference (*x*^2^ = 0.29, *P* = 0.588) between CBT-GSH (12.66%) and CAT-GSH (16.15%). This pattern was repeated at 24-weeks (*x*^2^ = 0.17, *P* = 0.683) and RCSC rates had increased for both interventions (i.e. 22.78% for CBT-GSH and 26.04% for CAT-GSH).

**Table 3:** Comparison of secondary outcomes related to service utilisation (attendance, drop-out and stepping-up rates) between CBT-GSH and CAT-GSH

	Total (*n* = 271)	CBT-GSH (*n* = 79)	CAT-GSH (*n* = 192)	Between- group comparison *P*
Total sessions, mean (s.d.)	4.48 (2.92)	3.57 (2.83)	4.86 (2.88)	**<0.001**
Attendance, *n* (%)				**0.005**
0 sessions	43 (15.9)	19 (24.1)*	24 (12.5)*	
1–2	41 (15.1)	15 (19.0)	26 (13.5)	
3–5	60 (22.1)	22 (27.8)	38 (19.8)	
6–8 (full)	127 (46.9)	23 (29.1)*	104 (54.2)*	
Dropped-out, *n* (%)	78 (28.8)	25 (31.6)	53 (27.6)	0.446
Stepped up, *n* (%)	57 (21.0)	16 (20.3)	41 (21.4)	0.840

Between-group differences are based on independent *t*-tests for continuous variables and chi-squared tests for categorical variables. *P-*values significant at <0.05 are highlighted in bold. Asterisk in the attendance outcomes denotes subset proportions that differ significantly (<0.05) from each other.

No serious or untoward incidents occurred in either arm. There were no significant differences between treatment arms for all secondary outcome measures in either the ITT sample or the complete-case sample at either follow-up (see Supplementary Appendix 5). When *post hoc* comparisons of return to treatment rates following trial participation were made between the interventions, there were no statistically significant differences between CBT-GSH and CAT-GSH (see Supplementary Appendix 7).

Overall pre–post-treatment mean change was −5.10 (s.d. = 6.17) on the GAD-7, −4.02 (s.d. = 5.88) on the PHQ-9 and −4.60 (s.d. = 9.33) on the WSAS for CBT-GSH versus −3.77 (s.d. = 5.18) on the GAD-7, −3.03 (s.d. = 5.22) on the PHQ-9 and −4.47 (s.d. = 8.15) on the WSAS for CAT-GSH.

There were no differences between the interventions (all *P* > 0.05) in terms of recovery, reliable recovery and reliable improvement rates and these were broadly in line with the service outcomes. There was a statistically significant and higher rate of deterioration on the GAD-7 in the CAT-GSH group (8%) relative to the CBT-GSH group (0%; *x^2^* = 4.58, *P* = 0.032). See Supplementary Appendix 8 for the graph benchmarking trial outcomes on the IAPT measures to routine service outcomes during the study period.

CAT-GSH participants were significantly more likely to start treatment, complete full treatment and attended significantly more sessions (i.e. CAT-GSH 4.86 sessions attended versus 3.57 sessions attended in CBT-GSH). There were no differences between CAT-GSH and CBT-GSH in rates of drop-out or subsequent stepping-up rates.

## Discussion

This trial found that CAT-GSH and CBT-GSH yielded largely similar anxiety outcomes on the BAI at 8- and 24-week follow-up, in treatments delivered with high indices of competence, equivalent levels of supervision and with no recorded adverse events. Trial outcomes were benchmarked as broadly equivalent to service outcomes, and effect sizes were also comparable with meta-analysed IAPT outcomes.^23^ Furthermore, RCSC rates improved over the follow-up period, suggesting that participants were continuing to make use of the GSH after treatment was completed. It is possible that one of the ways of countering the high rates of treatment attrition for GSH in IAPT^8^ is to routinely offer an informed choice of evidence-based GSH (i.e. that clearly differ in theoretical orientation and clinical approach, but not length, style and intensity). GSH offers scalability in services as these brief interventions can be delivered on the telephone^5^ as was the case here.

The patient preference design has enabled the first examination of patients’ preferences for different forms of GSH. An important finding was the high randomisation refusal rate; most participants clearly preferred to choose their treatment and may have otherwise refused participation unless offered this choice.^9^ Relatively little is known about how choice is offered and supported for psychological interventions in routine care. The single previous IAPT study showed that either there was no choice, alternatives to the first single choice were offered incrementally in response to patient resistance to the initial offer or the parallel presentation of multiple (potentially confusing) options.^8^ When choice is not informed, it is not really a choice at all, and becomes more of a ‘best guess’ on the part of the patient.

The preference results found that more participants chose CAT-GSH, and this preference was most likely when previous treatment episodes were CBT. There is meta-analytic evidence^24^ of the differential acceptability of CAT when it has been compared with other therapies in clinical trials and from practice-based evidence (odds ratio 0.67; 95% CI 0.48–0.93) and the current study found that CAT-GSH participants attended more sessions than CBT-GSH participants. It is acknowledged that it is difficult to disentangle effect of CAT-GSH and preference on increased attendance rates, as most CAT-GSH participants expressed a treatment preference. The psychoeducation that facilitated choice emphasised that CAT-GSH had a past–present focus, and this may have been attractive to participants seeking to better understand the origins of their anxiety, particularly for those that had previously received the explicitly ‘here and now' style of CBT in the service and it had been ineffective. It is worth noting that 16% across the arms did not attend a single GSH session (i.e., 24% in CBT-GSH and 13% in CAT-GSH). The treatments were delivered in a competent manner. Assessment of GSH competency has recently been automated using a system based on acoustic and linguistic features generated by an automatic speech recogniser to reduce time taken, as this has been acknowledged as a very time-consuming task for PWP clinical supervisors in routine practice.^25^

### Limitations

No health economic evaluation was conducted. As GSH approaches are common in IAPT as a way of enabling efficient throughput in the context of financial restrictions and high service demand, this would be an important future goal. The PRPPT had to be extended because of the differential preference rates between CAT-GSH and CBT-GSH and this meant that the CBT-GSH arm was underpowered. This extension in time meant that the CAT-GSH over recruited, as the pattern of differential preference then continued over the extension period. The comparability of the trial sample to a typical service population (i.e. typically 69.3% female and 28.8% from an ethnic minority background) is limited, as in the study participants the ethnic minority population was underrepresented. Lack of workbook translations likely contributed to underrepresentation and should be prioritised in future implementation efforts. Unequal sample sizes occurred because of the number of patients preferring CAT-GSH.

### Implications

This has been the first pragmatic PRPPT, to our knowledge, of different types of GSH interventions for anxiety and is important considering the sheer numbers now treated with GSH in routine services.^4,5^ Many more participants preferred CAT-GSH, but preference accommodation did not influence clinical outcomes. The broadly equivalent outcomes found between CAT-GSH and CBT-GSH indicates that CAT-GSH is an equally effective treatment option for the treatment of anxiety.

## Data Availability

The R code and the data are available on reasonable request from the corresponding author.

## Supplementary material

For supplementary material accompanying this paper visit https://doi.org/10.1192/bjp.2023.78. click here to view supplementary material
