# Cognitive-behavioural versus cognitive analytic guided self-help for mild-to-moderate anxiety: a pragmatic, randomised patient preference trial – CORRIGENDUM

**DOI:** 10.1192/bjp.2023.122

**Published:** 2023-11

**Authors:** Stephen Kellett, Charlotte Bee, Jess Smithies, Vikki Aadahl, Melanie Simmonds-Buckley, Niall Power, Caroline Dugen-Williams, Neil Fallon, Jaime Delgadillo

Keywords: Anxiety disorders; randomised controlled trial; patient choice; cognitive–analytic therapy; improving access to psychological therapies.

This article was originally published with author Caroline Dugen-Williams’ surname misspelled. The error has been corrected and the online PDF and HTML versions updated.
