# Supplementary material for: Cognitive–behavioural versus cognitive–analytic guided self-help for mild-to-moderate anxiety: a pragmatic, randomised patient preference trial
Source: Br J Psychiatry. 2023 Sep;223(3):438–45. doi: 10.1192/bjp.2023.78 (PMC10895510; doi:10.1192/bjp.2023.78)
Supplement: Supplementary file 1 [file S0007125023000788sup001.docx]

**Online Supplementary Appendices**

1. Protocol amendments after trial commencement
2. Sample description of randomised versus preference cohorts in each intervention arm
3. Exploratory analysis of preference effects
4. Baseline severity adjusted ANCOVA analyses of primary outcome (controlling for baseline severity only)
5. Full complete case analyses of primary and secondary outcomes
6. Sensitivity analyses using longitudinal multi-level modelling for primary outcome
7. Post-hoc analyses of treatment returners
8. Benchmarking of trial and service outcomes

**Appendix 1: Protocol amendments after trial commencement**

**Protocol Amendment Summary**

The major amendment once the trial had started was due to the COVID-19 pandemic when all the interventions needed to be delivered online. This was version 5 of the protocol after undergoing scientific and ethical reviews. Version 5 was ethically approved by the NHS Research Ethics Committee as a major Amendment to the Study Protocol as was consistent with all RCTs conducted in the UK during the pandemic.

| **Version Number** | **Date** | **Context or Reason** | **Changes Made** |
| --- | --- | --- | --- |
| 1 | Undated | First draft | None |
| 2 | 20/10/2018 | Scientific review | Secondary outcomes better specified. |
| 3 | 7/12/2018 | Following ethical review | Changes to patient information and communication with GPs. |
| 4 | 5/2/2019 | Scientific review | Improving specificity of outcome analysis in terms of timings and methods. |
| 5 | 29/1/2020 | COVID-19 | All interventions shifted to online delivery. |
| 6 | 30/3/2020 | Assessing previous service engagement and post-trial service engagement. | Collecting and assessing service engagement. |

**Appendix 2: Sample descriptions for allocation choice within each treatment arm**

Supplementary Table 1 reports the sample comparisons for the allocation choice (preference versus randomised cohorts) within in intervention arm. There were no significant differences in patient demographics, previous treatment history or baseline clinical characteristics according to allocation choice in either treatment arm. Due to unequal sample sizes and low power as a result of the high randomisation refusal rates, statistical comparisons should be viewed with caution.

**Supplementary Table 1.** *Baseline characteristics for the within-arm randomised versus preference cohorts for CBT-GSH and CAT-GSH conditions.*

|  | **CBT-GSH** | | **Between-group difference (p value)** | **CAT-GSH** | | **Between-group difference (p value)** |
| --- | --- | --- | --- | --- | --- | --- |
|  | **Randomised (n=8)** | **Preference (n=71)** |  | **Randomised**  **(n=11)** | **Preference (n=181)** |  |
| **Demographic characteristics** | | | | | | |
| Age | 39·62 (19·07) | 35·61 (12·76) | ·426 | 37·64 (14·73) | 37·01 (14·12) | ·887 |
| Sex |  |  | ·705 |  |  | ·698 |
| Male | 2 (25%) | 26 (37%) |  | 3 (27%) | 36 (20%) |  |
| Female | 6 (75%) | 45 (63%) |  | 8 (73%) | 145 (80%) |  |
| Ethnicity |  |  | 1·00 |  |  | 1·00 |
| White | 8 (100%) | 65 (92%) |  | 10 (91%) | 163 (90%) |  |
| Non-white | 0 (0%) | 5 (7%) |  | 1 (9%) | 18 (10%) |  |
| Missing | 0 (0%) | 1 (1%) |  | 0 (0%) | 0 (0%) |  |
| Employment status |  |  | ·348 |  |  | ·344 |
| Unemployed | 0 (0%) | 12 (17%) |  | 2 (18%) | 23 (13%) |  |
| Employed/Other | 8 (100%) | 59 (83%) |  | 7 (64%) | 155 (86%) |  |
| Missing | 0 (0%) | 0 (0%) |  | 2 (18%) | 3 (2%) |  |
| LTC |  |  | ·444 |  |  | 1·00 |
| Self-report LTC | 4 (50%) | 24 (34%) |  | 4 (36%) | 63 (35%) |  |
| No LTC | 4 (50%) | 47 (66%) |  | 7 (64%) | 118 (65%) |  |
| Taking medication |  |  | 1·00 |  |  | ·106 |
| Taking | 5 (63%) | 40 (56%) |  | 8 (73%) | 89 (49%) |  |
| Not taking | 3 (37%) | 28 (39%) |  | 2 (18%) | 84 (46%) |  |
| Missing | 0 (0%) | 3 (4%) |  | 1 (9%) | 8 (4%) |  |
| **Previous treatment history** | | | | | | |
| Previous treatment disclosed | 3 (38%) | 17 (24%) | ·410 | 4 (36%) | 91 (50%) | ·537 |
| Had CBT | 1 (13%) | 11 (16%) |  | 2 (18%) | 62 (34%) | ·342 |
| Had CAT | 0 (0%) | 0 (0%) |  | 0 (0%) | 3 (2%) | 1·00 |
| **Outcome measures** | | | | | | |
| BAI score | 32·75 (11·37) | 27·58 (10·12) | ·180 | 20·82 (12·58) | 25·50 (10·22) | ·101 |
| Mild | 0 (0%) | 10 (14%) | ·516 | 1 (9%) | 34 (19%) | ·721 |
| Moderate | 3 (38%) | 21 (30%) |  | 4 (36%) | 58 (32%) |  |
| Severe | 5 (63%) | 40 (89%) |  | 6 (55%) | 89 (49%) |  |
| GAD-7 score | 17·50 (3·66) | 16·79 (3·44) | ·583 | 16·55 (3·93) | 15·70 (4·01) | ·496 |
| PHQ-9 score | 16·88 (3·48) | 16·87 (5·17) | ·999 | 16·27 (4·34) | 14·86 (5·58) | ·410 |
| WSAS score | 20·50 (6·28) | 21·45 (7·56) | ·733 | 19·20 (10·49) | 19·63 (8·38) | ·875 |

Data are mean (SD) or n(%). Between-group differences are based on independent t-tests for continuous variables and chi-squared tests for categorical variables. CBT-GSH=Cognitive Behavioural Therapy-Guided Self-Help. CAT-GSH=Cognitive Analytic Therapy-Guided Self-Help. LTC=Long-term condition. BAI=Beck Anxiety Inventory. GAD-7=Generalised Anxiety Disorder-7. PHQ-9=Patient Health Questionnaire-9. WSAS=Work and social adjustment scale. *p* values significant at <·05 are highlighted in **bold**.

**Appendix 3: Baseline severity adjusted ANCOVA analyses of primary outcome (controlling for baseline severity only)**

Supplementary Table 2 presents the results of the primary analyses using analysis of covariance (ANCOVA) only adjusting for baseline severity (as stated in the protocol with no adjustments for other significantly different baseline covariates). Overall, there were no statistically significant differences in baseline severity adjusted mean BAI scores at 8-weeks or 24-weeks. When not controlling for significant differences between the intervention groups in baseline covariates, the non-significant mean difference at 8-week favoured CAT-GSH in contrast to the fully adjusted ANCOVA analyses (which favoured CBT-GSH; reported in the main manuscript). Both baseline-adjusted and fully adjusted non-significant mean differences favoured CAT-GSH at 24-week follow-up.

**Supplementary Table 2**. *BAI scores at post-treatment (8 weeks) and 24-week follow-up in the ITT and complete case samples adjusted for baseline severity*

|  | **CAT-GSH**  **N=192** | **CBT-GSH**  **N=79** | **ITT analysis**  **(observed and imputed data)^a^** | **Complete case analysis**  **(observed data only)^b^** |
| --- | --- | --- | --- | --- |
|  | **Mean BAI score^c^ (SE)** | **Mean BAI score^c^ (SE)** | **Adjusted between-group difference^d^ (95% CI)** | **Adjusted between-group difference^d^ (95% CI)** |
| 8-week post-treatment | 17·25 (0·57) | 17·50 (0·89) | -0·25 (-2·33 to 1·83) | 0·43 (-1·66 to 2·51) |
| 24-week follow up | 14·33 (0·52) | 15·07 (0·82) | -0·74 (-2·67 to 1·15) | -0·83 (-2·91 to 1·26) |

CAT-GSH: Cognitive Analytic Therapy-Guided Self-Help, CBT-GSH: Cognitive Behavioural Therapy-Guided Self-Help, ITT: Intention-to-treat, BAI: Beck Anxiety Inventory, SE: Standard error, CI: confidence interval.

^a^ITT sample based on missing outcome data imputed using missForest.

^b^Observed data based on n=84 in CAT-GSH and n=32 in CBT-GSH at 8 weeks; n=77 in CAT-GSH and n=33 in CBT-GSH at 24 weeks.

^c^Estimated marginal mean scores are reported for the ITT sample.

^d^Scores are adjusted for BAI baseline severity. Continuous covariates are mean centered.

**Appendix 4: Exploratory analysis of preference effects**

Supplementary Table 3 presents the between-group comparisons of BAI scores at 8-week and 24-week follow-up in the ITT sample for the randomised versus preference cohorts. At 8-weeks, there was a nonsignificant mean difference in BAI scores in favour of the preference cohort of -0·80 (-4·52 to 2·92; *p*=.672), corresponding to a small nonsignificant between-group Cohen’s *d* effect size for preference on outcomes (*d*=0·20, 95% CI -0·24 to 0·65). At 24-weeks, there was a nonsignificant mean difference in BAI scores in favour of the randomised cohort of (0·85; 95% CI -2·87 to 4·57; *p*=.626), corresponding to a small nonsignificant between-group Cohen’s *d* effect size for randomisation on outcomes (*d*=-0·21, 95% CI -0·24 to 0·65).

**Supplementary Table 3.**  *BAI scores at post-treatment (8 weeks) and 24-week follow-up in the ITT sample for the randomised and preference cohorts*

|  | **Randomised**  **N=19** | **Preference**  **N=252** | **ITT analysis**  **(observed and imputed data)^a^** | **ANCOVA model** |
| --- | --- | --- | --- | --- |
|  | **Mean BAI score^b^ (SE)** | **Mean BAI score^b^ (SE)** | **Adjusted between-group difference^c^ (95% CI)** |  |
| 8-week post-treatment | 18·11 (1·83) | 17·31 (0·55) | -0·80 (-4·52 to 2·92) | F(1, 267) = 0·18  *p*=·672 |
| 24-week follow up | 13·92 (1·68) | 14·77 (0·50) | 0·85 (-2·87 to 4·57) | F(1, 267) = 0·24  *p*=·626 |

ITT: Intention-to-treat, BAI: Beck Anxiety Inventory, SE: Standard error, CI: confidence interval.

^a^ITT sample based on missing outcome data imputed using missForest.

^b^Estimated marginal mean scores are reported for the ITT sample.

^c^Scores are adjusted for BAI baseline severity and treatment condition (CBT-GSH vs. CAT-GSH). Continuous covariates are mean centered.

Supplementary Table 4 reports the rates of attendance, drop-out and lost-to-follow-up rates for the randomised versus preference cohort in the overall sample and within intervention-arms. The overall pattern indicated a trend of a great number of sessions attended, higher rates of attendance and lower rates of drop-out and lost-to-follow-up for the preference relative to the randomised cohort. Statistical comparisons found only the improved attendance patterns for patients with a preference in the total sample and CAT-GSH group were significant.

**Supplementary Table 4.** *Effect of preference on intervention attendance and study retention*

|  | **CBT-GSH** | | **Between-groups comparison *p* value** | **CAT-GSH** | | **Between-groups comparison *p* value** | **Total** | | **Between-groups comparison *p* value** |
| --- | --- | --- | --- | --- | --- | --- | --- | --- | --- |
|  | **Randomised**  **(n=8)** | **Preference**  **(n=71)** |  | **Randomised**  **(n=11)** | **Preference (n=181)** |  | **Randomised**  **(n=19)** | **Preference**  **(n=252)** |  |
| Mean total sessions (SD) | 3·12 (2·36) | 3·62 (2·89) | ·596 | 3·27 (2·49) | 4·96 (2·88) | ·053 | 3·21 (2·37) | 4·58 (2·94) | ·079 |
| Attendance |  | | ·511 |  | | **·033** |  | | **·015** |
| 0 sessions | 1 (13%) | 18 (25%) |  | 1 (9%) | 23 (13%) |  | 2 (11%) | 41 (16%) |  |
| 1-2 | 3 (38%) | 12 (17%) |  | 5 (46%) | 21 (12%) |  | 8 (42%) | 33 (13%) |  |
| 3-5 | 3 (38%) | 19 (27%) |  | 1 (9%) | 37 (20%) |  | 4 (21%) | 56 (22% |  |
| 6+ | 1 (13%) | 22 (31%) |  | 4 (36%) | 100 (40%) |  | 5 (26%) | 122 (48%) |  |
| Dropped-out | 3 (38%) | 22 (31%) | 1·00 | 4 (36%) | 49 (27%) | ·748 | 7 (36%) | 71 (28%) | ·588 |
| Lost to follow-up | | | | | | | | |  |
| 8 week | 6 (75%) | 41 (57%) | ·574 | 8 (72%) | 100 (55%) | ·411 | 14 (74%) | 141 (56%) | ·206 |
| 24 week | 5 (63%) | 41 (58%) | 1·00 | 7 (64%) | 108 (60%) | 1·00 | 12 (63%) | 149 (59%) | ·918 |

Data are mean (SD) or n(%). Between-group differences are based on independent t-tests (within-treatment arm) and on analysis of covariance (ANCOVA) controlling for condition (total sample) for continuous variables and chi-squared tests for categorical variables. *p* values significant at <·05 are highlighted in **bold**.

**Appendix 5: Full complete case analyses of primary and secondary outcomes**

Supplementary Table 5 presents the full analyses for the primary and secondary outcomes in the complete case sample using observed data only.

**Supplementary Table 5**. *Primary and secondary outcomes at post-treatment (8 weeks) and 24-week follow-up in the complete case sample*

|  | **CAT-GSH**  **N=84^a^** | **CBT-GSH**  **N=32^a^** | **Complete case analysis**  **(observed data only)** |
| --- | --- | --- | --- |
|  | **Mean symptom score (SE)** | **Mean symptom score (SE)** | **Adjusted between-group difference^b^ (95% CI)** |
| BAI |  |  |  |
| 8-week post-treatment | 16·11 (2·59) | 15·54 (2·74) | 0·57 (-3·81 to 4·94) |
| 24-week follow up | 10·79 (2·48) | 12·07 (2·69) | -1·28 (-5·41 to 2·85) |
| GAD-7 |  |  |  |
| 8-week post-treatment | 9·13 (1·36) | 8·64 (1·44) | 0·49 (-1·80 to 2·78) |
| 24-week follow up | 7·85 (1·18) | 8·23 (1·26) | -0·38 (-2·67 to 1·91) |
| PHQ-9 |  |  |  |
| 8-week post-treatment | 10·08 (1·52) | 9·20 (1·60) | 0·87 (-1·70 to 3·44) |
| 24-week follow up | 8·73 (1·22) | 8·98 (1·31) | -0·25 (-2·62 to 2·13) |
| WSAS |  |  |  |
| 8-week post-treatment | 13·94 (2·64) | 11·69 (2·71) | 2·25 (-1·83 to 6·32) |
| 24-week follow up | 13·46 (2·11) | 13·26 (2·18) | 0·21 (-3·63 to 4·05) |

CAT-GSH: Cognitive Analytic Therapy-Guided Self-Help, CBT-GSH: Cognitive Behavioural Therapy-Guided Self-Help, BAI: Beck Anxiety Inventory, GAD-7=Generalised Anxiety Disorder-7, PHQ-9=Patient Health Questionnaire-9, WSAS=Work and social adjustment scale, SE: Standard error, CI: confidence interval.

^a^Observed data based on n=84 in CAT-GSH and n=32 in CBT-GSH at 8 weeks; n=77 in CAT-GSH and n=33 in CBT-GSH at 24 weeks.

^b^Scores are adjusted for BAI baseline severity, allocation choice (preference vs. randomised), sex, previous treatment, GAD-7 baseline severity and PHQ-9 baseline severity. Analysis of WSAS outcomes are also adjusted for WSAS baseline severity· Continuous covariates are mean centered.

**Appendix 6: Sensitivity analyses using longitudinal multi-level modelling**

Supplementary Table 6 provides the full coefficient results of the longitudinal multilevel modelling for the effect of CBT-GSH and CAT-GSH on BAI scores from screening, post-treatment and follow-up in the full intention-to-treat sample (using observed data only). The model controls for baseline covariates identified as significantly associated with treatment received or allocation choice as adjusted for in the ANCOVA analyses reported in the main manuscript (baseline GAD-7, baseline PHQ-9, sex, had previous treatment and allocation choice). The primary hypothesis test of the treatment group*time interaction on BAI scores was not significant indicating the trajectory of BAI scores did not differ between interventions, supporting findings from the primary ANOCVA analysis.

**Supplementary Table 6**. *Means, effect sizes and longitudinal multi-level modelling results for CBT-GSH and CAT-GSH on the primary outcome (BAI) in the ITT sample (using observed data only)*

|  | **CBT-GSH**  **(n=79)^a^** | | **CAT-GSH**  **(n=192)^a^** | | **Between-group ES (95% CI)** | |
| --- | --- | --- | --- | --- | --- | --- |
| BAI scores | **Mean (SD)** | | **Mean (SD)** | |  |  |
| Screening (week 0) | 28·10 (10·30) | | 25·81(10·41) | | -0·22 (-0·48 to 0·04) | |
| Post-treatment (week 8) | 15·56 (10·51) | | 16·01 (10·92) | | 0·04 (-0·37 to 0·45) | |
| Follow-up (week 24) | 14·76 (12·02) | | 13·44 (10·09) | | -0·12 (-0·53 to 0·28) | |
|  | **Longitudinal multilevel model results^a^** | | | | | |
|  | ***B* estimate** | **SE** | | **95% CI** | | ***p*^d^** |
| Treatment effect |  |  | |  | |  |
| Intercept | 28·17 | 1·17 | | 25·86 to 30·48 | | **<·001** |
| Time - loglinear (weeks)^b^ | -4·67 | 0·59 | | -5·84 to -3·49 | | **<·001** |
| Treatment group (ref: CAT-GSH) | -0·463 | 0·65 | | -1·74 to 0·82 | | ·478 |
| Time*treatment group | 0·052 | 0·32 | | -0·58 to 0·68 | | ·872 |
| Covariate main effect^c^ |  |  | |  | |  |
| Baseline GAD-7 | 0·61 | 0·18 | | 0·25 to 0·97 | | **·001** |
| Baseline PHQ-9 | 0·45 | 0·13 | | 0·19 to 0·71 | | **<·001** |
| Sex (ref: female) | 0·74 | 0·67 | | -0·58 to 2·05 | | ·269 |
| Previous treatment (ref: had treatment) | 0·57 | 0·59 | | -0·59 to 1·73 | | ·336 |
| Allocation choice (ref: preference) | -2·22 | 1·11 | | -4·40 to -0·03 | | **·047** |
| Covariate interactions |  |  | |  | |  |
| Time*baseline GAD-7 | -0·22 | 0·09 | | -0·39 to -0·04 | | **·014** |
| Time*baseline PHQ-9 | -0·00 | 0·07 | | -0·13 to 0·13 | | ·963 |
| Time*sex | 0·35 | 0·34 | | -0·32 to 1·02 | | ·300 |
| Time*previous treatment | -0·00 | 0·29 | | -0·58 to 0·57 | | ·991 |
| Time*allocation choice | 0·65 | 0·58 | | -0·50 to 1·81 | | ·264 |

CAT-GSH: Cognitive Analytic Therapy-Guided Self-Help, CBT-GSH: Cognitive Behavioural Therapy-Guided Self-Help, BAI: Beck Anxiety Inventory, ES: Cohen’s *d* effect size*, B:* regression coefficient, SE; standard error, 95% CI: confidence intervals*,* GAD-7: Generalised Anxiety Disorder-7, PHQ-9: Patient Health Questionnaire-9, WSAS: Work and social adjustment scale, ref: reference category.

^a^Based on Intention-to-treat (ITT) using observed data from all N=271 patients allocated to an intervention; Week 0; CBT-GSH N=79, CAT-GSH N=192, Week 8: CBT-GSH N=32, CAT-GSH N=84, Week 24; CBT-GSH N=33, CAT-GSH N=77.

^b^Time based on three data collection points at weeks 0, 8 and 24 and converted to a loglinear scale.

^c^Continuous covariates are mean centered.

*^d^p* values significant at <·05 are highlighted in **bold**.

**Appendix 7: Post-hoc analyses of treatment returners**

Supplementary Table 7 presents post-hoc comparisons of treatment returner rates between interventions. There were no statistically significant differences between groups.

**Supplementary Table 7.**  Analysis of treatment returners during the trial

|  | **Total**  **(N=271)** | **CBT-GSH**  **(N=79)** | **CAT-GSH**  **(N=192)** | **Between-groups comparison (*p* value^a^)** |
| --- | --- | --- | --- | --- |
| Returned for treatment | 32 (11·8%) | 13 (16·5%) | 19 (9·9%) | ·128 |
| Mean time in months between episodes (SD) | 7·50 (5·13) | 9·42 (5·33) | 6·31 (4·87) | ·110 |
| Treatment on return |  |  |  | ·245 |
| LI CBT | 11 (4·1%) | 5 (6·3%) | 6 (3·1%) |  |
| HI CBT | 7 (2·6%) | 4 (5·1%) | 3 (1·6%) |  |
| Counselling | 8 (2·9%) | 1 (1·3%) | 7 (3·6%) |  |
| Awaiting assessment/other referral | 6 (2·2%) | 3 (3·8%) | 3 (1·5%) |  |
| Returners who had previous treatment | 15 (46·8%) | 38.5% (5) | 52.6% (10) | ·430 |
| Post-tx recovery status | N=32 | N=13 | N=19 | ·643 |
| Reliable improvement | 19 (59·4%) | 7 (53·8%) | 12 (63·1%) |  |
| Reliable deterioration | 1 (3·1%) | 0 (0%) | 1 (5·6%) |  |
| Post-tx caseness |  |  |  |  |
| GAD-7 caseness | 19 (59·4%) | 8 (61·5%) | 11 (57.9%) | ·757 |
| PHQ-9 caseness | 17 (53·1%) | 8 (61·5%) | 9 (47.4%) | ·367 |

Data are mean (SD) or n (%). Between-group differences are based on independent t-tests for continuous variables and chi-squared tests for categorical variables. *p* values significant at <·05 are highlighted in **bold**. Asterisk (*) denotes subset proportions that differ significantly from each other.

**Appendix 8: Benchmarking of trial and service outcomes**

**Supplementary Figure 1.** *Pre-Post Treatment IAPT GAD-7 and PHQ-9 Recovery Metrics For CBT-GSH and CAT-GSH Interventions*

*Note:* Recovery rates are based on IAPT definition of patients who have received a course of treatment (>= 2 treatment sessions); CBT-GSH N=55 and CAT-GSH N=154.
